# Evolution of Light-Sensitive Proteins in Optogenetic Approaches for Vision Restoration: A Comprehensive Review

**DOI:** 10.3390/biomedicines13020429

**Published:** 2025-02-10

**Authors:** Kamil Poboży, Tomasz Poboży, Paweł Domański, Michał Derczyński, Wojciech Konarski, Julia Domańska-Poboża

**Affiliations:** 1 Department of Neurosurgery, Brodnowski Masovian Hospital, 03-242 Warsaw, Poland; pobozykamil@gmail.com; 2 Department of Orthopedic Surgery, Ciechanów Hospital, 06-400 Ciechanów, Poland; pawel_domanski@op.pl; 3 Independent Researcher, 05-123 Olszewnica Stara, Poland; michalderczynski@gmail.com; 4 Medical Rehabilitation Center, 05-120 Legionowo, Poland; wkonarski@poczta.onet.pl; 5 Department of Rheumatology, National Institute of Geriatrics, Rheumatology and Rehabilitation, 02-637 Warsaw, Poland; julia-domanska03@wp.pl

**Keywords:** retinal degeneration, optogenetics, opsin, channelrhodopsin-2, ChrimsonR, multi-characteristic opsin 1

## Abstract

Retinal degenerations, such as age-related macular degeneration and retinitis pigmentosa, present significant challenges due to genetic heterogeneity, limited therapeutic options, and the progressive loss of photoreceptors in advanced stages. These challenges are compounded by difficulties in precisely targeting residual retinal neurons and ensuring the sustained efficacy of interventions. Optogenetics offers a novel approach to vision restoration by inducing light sensitivity in residual retinal neurons through gene delivery of light-sensitive opsins. This review traces the evolution of opsins in optogenetic therapies, highlighting advancements from early research on channelrhodopsin-2 (ChR2) to engineered variants addressing key limitations. Red-shifted opsins, including ReaChR and ChrimsonR, reduced phototoxicity by enabling activation under longer wavelengths, while Chronos introduced superior temporal kinetics for dynamic visual tracking. Further innovations, such as Multi-Characteristic Opsin 1 (MCO1), optimized opsin performance under ambient light, bridging the gap to real-world applications. Key milestones include the first partial vision restoration in a human patient using ChrimsonR with light-amplifying goggles and ongoing clinical trials exploring the efficacy of opsin-based therapies for advanced retinal degeneration. While significant progress has been made, challenges remain in achieving sufficient light sensitivity for functional vision under normal ambient lighting conditions in a manner that is both effective and safe, eliminating the need for external light-enhancing devices. As research progresses, optogenetic therapies are positioned to redefine the management of retinal degenerative diseases, offering new hope for millions affected by vision loss.

## 1. Introduction

Retinal degenerations (RDs), such as age-related macular degeneration (AMD) and retinitis pigmentosa (RP), are common forms of neurodegenerative diseases, characterized by genetic and clinical heterogeneity, making diagnosis and treatment challenging [1]. These degenerative disorders often present with symptoms like night blindness, tunnel vision, and progressive vision loss, which may eventually lead to complete blindness. Mutations in over 300 genes have been implicated in inherited retinal diseases, further complicating diagnosis, as different mutations can lead to similar phenotypes, and the same mutation can result in varied clinical outcomes even within the same family [2]. The heterogeneity of retinal dystrophies also extends to disease progression and symptom onset, with different subtypes presenting distinct patterns of peripheral and central vision decline [3]. Despite the lack of effective treatments for most retinal dystrophies, recent advances in gene therapy, particularly the approval of Luxturna for RPE65-associated retinal dystrophies, offer new hope for therapeutic intervention, with several ongoing clinical trials targeting other subtypes [4].

Despite pathological remodeling in the retina due to photoreceptor loss, bipolar cells (BCs), which relay visual signals from photoreceptors to retinal ganglion cells (RGCs), and RGCs, which transmit these signals to the brain, remain intact even in advanced stages of inherited RDs [5,6,7,8,9,10,11]. Optogenetics is a technique that enables the modulation of neuronal activity using light. In recent years, advancements in optogenetic technology have created new possibilities for understanding and treating diseases such as Parkinson’s disease, epilepsy, and arrhythmias [12,13,14].

**Figure 1:**
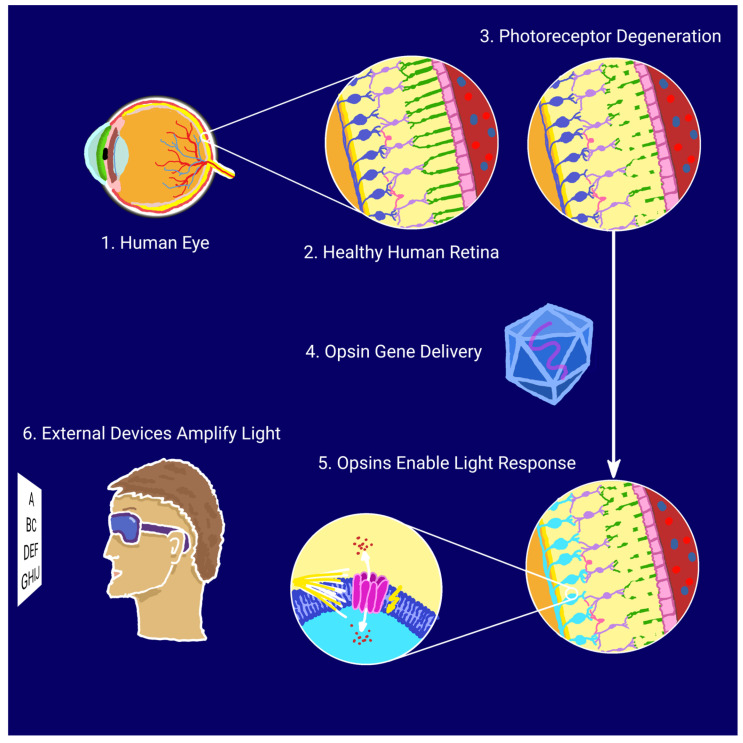
Schematic illustration of optogenetic vision restoration.

Optogenetics enables the precise control of neuronal activity through light-responsive proteins, or opsins, delivered to target cells via gene therapy. In retinal disease models, adeno-associated viruses (AAVs), characterized by low immunogenicity and sustained gene expression, are the vectors of choice, with intravitreal or subretinal injections most commonly used for delivery [15]. Numerous studies indicate that optogenetics could become a breakthrough in restoring partial vision for individuals with retinal degenerations; by rendering surviving neurons in the visual pathway—such as bipolar or ganglion cells—light-sensitive, it is possible to bypass the photoreceptor cells damaged by disease and, consequently, restore the function of the visual pathway [16,17]. The optogenetic approach for vision restoration is illustrated schematically in Figure 1.

Over the years, the repertoire of available opsins has expanded significantly, with variants engineered to produce distinct neuronal effects (depolarization, hyperpolarization, or complex signaling modulation), operate through specific mechanisms (ion channel or signaling opsins), and respond to finely tuned light parameters [18,19]. A primary focus is optimizing opsins with low light intensity thresholds to mitigate phototoxicity [20]. Red-shifted opsins, responsive to light wavelengths around 500–600 nm, are often preferred due to their favorable phototoxicity profile. This advantage arises because red-shifted light penetrates biological tissues more efficiently and scatters less than shorter wavelengths, such as blue or green light, reducing the need for high-intensity illumination that could damage retinal cells. Additionally, red-shifted opsins minimize thermal effects and oxidative stress, both of which are linked to phototoxicity, ensuring safer and more sustainable stimulation of the retina. Adjustments to activation duration are tailored to facilitate seamless tracking of dynamic environments [21].

Parallel advancements are enhancing the efficacy of optogenetic therapy in vision restoration. These include the refinement of light-amplifying goggles, the exploration of compact optical implants for precise light guidance, and the integration of optogenetics with adjunctive therapies, such as retinal prosthetics, stem cell therapies, and gene therapies supporting retinal cell viability [22,23,24].

Given the numerous challenges in treating retinal degenerations and the promising advancements in optogenetics, this review aims to provide a comprehensive examination of the evolution of light-sensitive proteins used in optogenetic approaches for vision restoration. The objective is to elucidate how innovations in opsin engineering have created new avenues for treating vision loss, particularly by targeting residual retinal cells that remain functional despite photoreceptor degeneration. By analyzing the properties, applications, and clinical potential of various opsins, this review will offer insights into current progress and highlight future directions for enhancing optogenetic therapies as a viable solution for patients with degenerative retinal diseases.

## 2. Microbial Opsins

### 2.1. Microbial Depolarizing Opsins

#### 2.1.1. ChR2 (Channelrhodopsin-2)

Channelrhodopsin-2 (ChR2), derived from the unicellular green alga Chlamydomonas reinhardtii, marked a pioneering step in optogenetic vision restoration. The seminal study by Bi et al. (2006) demonstrated that AAV-mediated ChR2 expression in retinal cells, predominantly retinal ganglion cells (RGCs), of photoreceptor-deficient mice partially restored visually evoked potentials (VEPs). This established ChR2’s ability to bypass degenerated photoreceptors and directly confer light sensitivity to surviving retinal neurons [25]. Subsequent investigations in dystrophic RCS (Royal College of Surgeons) rats corroborated these findings, showing restoration of visual functions through optokinetic responses and VEPs, even in the context of advanced retinal degeneration [26,27].

Targeted expression of ChR2 in ON bipolar cells, achieved via a cell-specific promoter, further highlighted its potential. In the rd1 (retinal degeneration 1) mouse model of retinal degeneration, ChR2-transduced ON bipolar cells exhibited light-driven neuronal activity, effectively relaying signals to downstream ganglion cells and the visual cortex. This resulted in significant improvements in vision-dependent behavioral tasks [28]. Additional studies on the method targeting ON bipolar cells confirmed the safety, long-term efficacy, and physiological restoration of ON responses in postsynaptic ganglion cells, along with measurable enhancements in visually guided behaviors [29,30].

Clinical translation of ChR2-based therapies has shown promising outcomes. A phase I/II trial (ID NCT02556736) involving 14 adult patients with advanced retinitis pigmentosa reported no severe adverse effects within six months of intravitreal administration of an AAV2 (adeno-associated virus 2) vector encoding ChR2. These results provide critical evidence supporting the safety and feasibility of ChR2-based optogenetic interventions for human retinal degenerations.

Despite its revolutionary role, ChR2 has certain limitations, including relatively high light intensity requirements and suboptimal response kinetics, which can impact its efficacy and clinical applicability. These challenges have spurred the development of enhanced opsins engineered to address these shortcomings.

#### 2.1.2. CatCh (Calcium-Translocating Channelrhodopsin)

Following the foundational work on ChR2, advancements in opsin engineering led to the development of CatCh, a calcium-translocating channelrhodopsin variant that significantly enhanced light sensitivity. Kleinlogel et al. (2011) introduced CatCh as a modified form of ChR2, engineered to allow improved calcium permeability, which in turn elevated the membrane potential and facilitated the activation of downstream sodium channels. This modification yielded a 70-fold increase in light sensitivity and accelerated response kinetics compared to wild-type ChR2. Thus, CatCh allowed for neuronal activation at much lower light intensities, minimizing the risk of phototoxicity and supporting potential clinical applications where lower light intensities are desirable [31]. Further studies demonstrated the efficacy of CatCh in RGCs of macaque models, achieving substantial light-induced activity at safe light levels [32].

#### 2.1.3. PsCatCh2.0 (Platymonas Subcordiformis Channelrhodopsin 2.0)

The evolution of optogenetic tools continued with the development of PsCatCh2.0 (Platymonas subcordiformis Channelrhodopsin 2.0), an advanced iteration derived from the highly efficient channelrhodopsin PsChR originally isolated from Platymonas subcordiformis. PsChR is a blue-shifted channelrhodopsin with approximately threefold higher unitary conductance and greater sodium selectivity compared to ChR2 [33]. PsCatCh2.0 further enhances PsChR’s calcium and sodium conductance and optimizes it for retinal ganglion cell targeting. PsCatCh2.0 showed significant improvements in light sensitivity, requiring 100 times less light intensity to elicit photocurrents comparable to CatCh. It also demonstrated rapid kinetics, allowing it to track light pulses up to 32 Hz without desensitization, a critical feature for high temporal resolution in visual restoration. In vivo experiments in blind rodent models confirmed PsCatCh2.0’s capacity to restore visual behaviors and evoke activity in the visual cortex, supporting its potential as an effective tool for future clinical applications in vision restoration [34].

#### 2.1.4. VChR1 (Volvox Channelrhodopsin-1)

The discovery of Volvox channelrhodopsin-1 (VChR1) from Volvox carteri provided a significant advancement in optogenetics due to its red-shifted absorption spectrum. With a peak sensitivity near 540 nm (compared to approximately 470 nm for ChR2), VChR1 allows for deeper tissue penetration and reduces the risk of phototoxicity compared to blue-light opsins, making it an appealing candidate for vision restoration in retinal degenerations. Zhang et al. (2008) demonstrated that VChR1 could effectively stimulate neurons under yellow light, enabling excitation in wavelengths less harmful to retinal cells [35].

#### 2.1.5. ReaChR (Red-Activatable Channelrhodopsin)

While VChR1 exhibited strong photoresponsiveness, it faced challenges in membrane trafficking and expression in mammalian systems, limiting its practical application [36]. To address these limitations, ReaChR (Red-activatable Channelrhodopsin) was developed as an enhanced red-shifted variant. Lin et al. (2013) successfully engineered ReaChR with improved membrane localization, trafficking, and a further red-shifted peak around 590–630 nm, enabling effective activation by red light. ReaChR’s design made it suitable for deep retinal stimulation, bypassing the need for invasive light delivery methods [37]. This innovation paved the way for therapeutic applications, as demonstrated by Sengupta et al. (2016), who showed that ReaChR could restore light sensitivity in animal models at intensities safe for the human retina, with functional responses observed at both cortical and behavioral levels [38]. Furthermore, ReaChR-reactivated RGCs maintain small receptive fields, ensuring high spatial resolution [39].

#### 2.1.6. bReaChES

Further refinement led to bReaChES, a variant of ReaChR with enhanced light sensitivity, temporal precision, and peak responsiveness around 570–590 nm [40,41]. The opsin demonstrated functional improvements in murine models of retinal degeneration, achieving consistent responses under ambient lighting and maintaining flicker tracking up to 50 Hz, approaching the temporal resolution of human photopic vision [40,41].

#### 2.1.7. mVChR1 (Modified VChR1)

Alongside the development of ReaChR and bReaChES to address the demand for red-shifted opsins, significant progress was also achieved with mVChR1, a chimeric opsin derived from VChR1 and Chlamydomonas reinhardtii opsin 1 (ChR1). Tomita et al. (2014) developed mVChR1 to achieve a broader light sensitivity range while overcoming VChR1’s challenges in membrane trafficking and expression. By integrating structural elements from ChR1, mVChR1 exhibited improved membrane localization and functioned effectively across wavelengths from 468 to 640 nm. In vivo studies demonstrated that mVChR1 could restore VEPs and behavioral responses in blind rat models, providing stable, long-term functionality under broad-spectrum light conditions, making it a promising candidate for clinical translation [42].

#### 2.1.8. ComV1 (ex3mV1)

Building on mVChR1, Watanabe et al. (2021) introduced ComV1, an optimized opsin with enhanced sensitivity to daylight conditions [43]. Through targeted modifications in extracellular loops (notably ex3), ComV1 demonstrated improved conductance and a significant reduction in the required light intensity for activation compared to mVChR1. This modification allowed ComV1 to elicit reliable visual responses under μW/mm^2^ light levels, advancing the potential for non-invasive therapeutic applications [43]. Further refinements led to the H172A variant of ComV1, designed to optimize channel kinetics by modifying a single amino acid in the transmembrane domain. This variant enhanced the temporal resolution and photocurrent stability without compromising light sensitivity, reinforcing ComV1’s suitability for high-performance vision restoration therapies [44].

Together, these red-shifted opsins highlight a progressive enhancement in optogenetic tools, addressing both safety and functional efficacy requirements for vision restoration therapies.

#### 2.1.9. CoChR (Chloromonas Oogama Channelrhodopsin)

In parallel with advancements in red-shifted and broad-range channelrhodopsins, researchers also explored Chloromonas oogama channelrhodopsin (CoChR). CoChR, a blue-light-sensitive opsin with high light sensitivity and a large photocurrent amplitude, emerged as a potential candidate for neural activation with minimal phototoxicity [45]. In subsequent studies, CoChR’s robust expression and responsiveness to blue wavelengths allowed it to be integrated into Caenorhabditis elegans and mouse models for vision restoration, where it effectively activated neurons under low light intensities [45]. However, studies also recognized the persisting need for further improvements in light sensitivity in ambient lighting conditions [46,47]. This led Ganjawala et al. (2019) to engineer CoChR mutants—CoChR-LC (L112C mutation) and CoChR-3M (H94E/L112C/K264T mutations)—which demonstrated significantly enhanced light sensitivity [48]. These modifications extended CoChR’s capacity to restore vision in blind mouse models even under ambient light, achieving notable improvements in contrast sensitivity and visual acuity. This series of developments refined CoChR’s application in optogenetic vision restoration, providing a tool optimized for high efficacy under ambient light conditions and aligning with practical therapeutic requirements [48].

#### 2.1.10. Chronos (ShChR, Stigeoclonium Helveticum Channelrhodopsin)

The identification of Chronos, an ultra-fast channelrhodopsin derived from Stigeoclonium helveticum, marked a notable advance in optogenetics due to its improved kinetics and light sensitivity. Chronos is a blue-light-activated opsin with faster on- and off-kinetics than previously developed opsins, making it highly suitable for applications requiring precise temporal control [45]. Recent advancements have highlighted Chronos’s potential in restoring visual function under clinically viable conditions. Using an AAV2 vector for targeted delivery to retinal ganglion cells, Chronos was shown to produce robust and reliable VEPs in animal models at lower light intensities, further minimizing phototoxic risk. The stability and consistency of Chronos-driven responses under varying light conditions underscored its efficacy as an optogenetic protein for vision restoration. These findings position Chronos as an essential opsin, providing high temporal resolution and safety necessary for future clinical translation [49]. The clinical potential of Chronos is further being explored in an ongoing phase 1/2 clinical trial (ID NCT04278131). This non-randomized, open-label, dose-escalation study investigates BS01, a non-replicating, rep-/cap-deleted recombinant AAV vector designed to express ChronosFP (Chronos fused to green fluorescent protein) gene. The trial aims to evaluate the safety and efficacy of BS01 in patients with retinitis pigmentosa, offering critical insights into the therapeutic application of Chronos-based optogenetic strategies for vision restoration.

#### 2.1.11. ChrimsonR (CrimsonR, CnChR1, Chlamydomonas Noctigama Channelrhodopsin 1)

The development of ChrimsonR, a red-light-activated opsin, represented a crucial step forward in optogenetics, ultimately paving the way for groundbreaking human applications. Chrimson presents a peak activation wavelength around 590 nm, enabling its safe and effective use in therapeutic settings [45].

Cheong et al. (2018) explored the potential of ChrimsonR for optogenetic vision restoration by combining it with high-resolution adaptive optics imaging, allowing precise, all-optical stimulation and recording of retinal neurons in vivo [50]. In their study on retinal degeneration in the rd10 mouse model, ChrimsonR-expressing ganglion cells demonstrated robust and sustained responses to red light, with functional visual responses observed over an extended period. This study underscored ChrimsonR’s stability and efficacy in vivo, offering direct insights into its functional integration in the retina. The use of red-shifted light minimized phototoxic effects while maintaining visual response consistency, enhancing the clinical viability of ChrimsonR for sustained vision restoration applications [50]. Further studies demonstrated that ChrimsonR expressed in retinal ganglion cells could restore light sensitivity in blind macaque retinas, with responses maintained over time and transmitted effectively to the visual cortex [51,52]. These studies confirmed that ChrimsonR-transduced ganglion cells could achieve sufficient spatial and temporal resolution to support vision-related tasks, presenting it as a robust candidate for human clinical trials.

A study by Sahel et al. (2021) demonstrated the first partial restoration of visual function in a blind patient with advanced retinitis pigmentosa using an optogenetic approach with ChrimsonR, combined with wearable light-stimulating goggles [53]. The patient participated in the phase I/IIa PIONEER clinical trial (ID NCT03326336). ChrimsonR was administered intravitreally and transduced into retinal ganglion cells through an AAV2 vector. After undergoing a structured training regimen, the patient regained the ability to perceive and interact with objects, marking a groundbreaking milestone in the clinical application of optogenetics for vision restoration. The light-stimulating goggles, designed to project amber light (595 nm) onto the retina, were critical for enhancing visual contrast and facilitating object recognition in real-world scenarios. The visual training program included exercises aimed at improving the patient’s ability to align their gaze with the light-stimulation beam and adapt to object recognition tasks. These exercises were pivotal in enabling the patient to interpret visual input effectively, highlighting the importance of rehabilitation protocols in optogenetic therapy. In real-world settings, the patient reported significant improvements, such as identifying crosswalks and objects in their environment. These findings provide strong evidence for the translational potential of ChrimsonR in restoring meaningful visual function in blind patients [53].

Later studies on living primates have shown that the ChrimsonR-driven RGCs’ responses demonstrate patterns similar to photoreceptor-mediated activity, underscoring ChrimsonR’s ability to mimic natural visual processing pathways [54].

Collectively, these advances solidify ChrimsonR’s role as a powerful tool in optogenetic vision restoration, combining red-shifted activation with the high spatial fidelity and stability necessary for effective and safe therapeutic applications. Its selection for clinical use is justified by its compatibility with human retinal physiology, minimized phototoxicity, and integration with wearable technologies designed for vision enhancement.

#### 2.1.12. ChRmine

The identification of ChRmine, a pump-like channelrhodopsin derived from Rhodomonas lens, revealed an opsin offering exceptionally high photocurrent and a red-shifted activation profile. ChRmine elicits robust neuronal activation with minimal light intensity, achieving precise spatiotemporal control of large cortical volumes [55]. An assessment of ChRmine’s structural properties via cryo-EM identified key adaptations such as a trimeric assembly and modifications in transmembrane regions, which support both high sensitivity and a broad activation spectrum. These properties allow for ChRmine’s enhanced red-light responsiveness, crucial for safer and effective activation in retinal applications. Leveraging this structure, researchers developed variants such as hsChRmine with faster kinetics, frChRmine with increased red-light sensitivity, and rsChRmine optimized for reduced blue-light interference, further enhancing ChRmine’s usability across diverse lighting environments [56]. Recently, Bansal et al. (2024) confirmed that ChRmine and its variants could sustain high-frequency spiking under broadband light sources, making them suitable for ambient or indoor settings without requiring external light-amplifying devices. ChRmine’s comprehensive activation spectrum and minimal phototoxicity present a substantial advancement in optogenetic vision restoration, offering patients a safer, effective means of regaining functional vision under natural lighting [57].

#### 2.1.13. White-Opsin

The creation of white-opsin represents an innovative approach to opsin development for optogenetic vision restoration under ambient light conditions. Instead of relying on a single opsin, Batabyal et al. (2015) engineered white-opsin by fusing three distinct opsins—ChR2, C1V1, and ReaChR—each responsive to different parts of the visible spectrum: blue, green, and red wavelengths, respectively [58]. This broad-spectrum sensitivity enables white-opsin-sensitized cells to generate high photocurrents under white light, significantly enhancing light sensitivity compared to narrow-band opsins. It is worth noting that some of the previously mentioned opsins also function across a wide spectrum, but the method of combining different opsins to sum their spectral responses presents an exciting direction for optogenetics. The fusion protein displayed an approximately 30-fold increase in photocurrent compared to ChR2 under similar conditions, providing sufficient stimulation with ambient light, thus circumventing the need for high-intensity, narrow-band illumination. This broadband sensitivity also allowed white-opsin to lower the activation threshold, reducing the phototoxic risk and eliminating the need for external light-amplifying devices [58].

#### 2.1.14. MCO1 (Multi-Characteristic Opsin 1)

The development of the Multi-Characteristic Opsin 1 (MCO1) marked a major advance in optogenetic tools aimed at functional vision restoration for retinal degeneration. First introduced by Wright et al. in 2017, MCO1 was designed to be activatable under ambient light, expanding the usability of optogenetic therapies beyond high-intensity light conditions that pose a phototoxicity risk [59,60]. MCO1 combines distinct characteristics from various opsins to achieve broad-spectrum sensitivity, facilitating its activation across a range of wavelengths and significantly reducing the energy required for stimulation. This design makes MCO1 especially effective for practical applications, as demonstrated in preclinical studies with retinal degeneration models, where MCO1 showed strong, stable expression and robust visually guided behaviors in treated animals [59,60].

Subsequent studies demonstrated that MCO1-transduced retinal cells retained functional stability and structural integrity over time. The use of AAV2-mediated delivery ensured long-lasting opsin expression in retinal bipolar cells, where MCO1 activation under ambient light enabled meaningful improvements in visual acuity and optomotor responses in mouse models [61,62,63]. These findings were pivotal in translating MCO1 into clinical trials, as evidenced by the promising early outcomes in human studies in patients with severe vision loss due to retinal degenerative diseases like retinitis pigmentosa and Stargardt disease [64,65,66,67].

Recent clinical trials have explored the potential of optogenetic therapies using MCO1 delivered via intravitreal AAV2 vectors to improve visual function in patients with severe retinal degenerative conditions. Two pivotal studies, the RESTORE study (ID NCT04945772) and the STARLIGHT trial (ID NCT05417126), have provided valuable insights into the efficacy and safety of this approach.

The RESTORE study focused on patients with retinitis pigmentosa and investigated the use of MCO1 to enhance best-corrected visual acuity (BCVA). Over a 12-month period, statistically significant gains in BCVA were observed, demonstrating the therapy’s ability to restore vision in individuals with this degenerative condition [64,65].

In the STARLIGHT phase 2 trial, multi-characteristic opsin was evaluated in patients with Stargardt disease. The six-month results showed improvements in BCVA alongside a consistent safety profile, indicating that this approach could address the therapeutic needs of individuals with this progressive condition [66]. Longitudinal data over 48 weeks further validated these findings, with patients using wearable magnifiers achieving up to a 31-letter improvement in BCVA. Importantly, no severe adverse effects were reported, underscoring MCO1’s potential as a safe and effective treatment for retinal degenerative diseases [67].

Together, these trials highlight the promise of AAV2-delivered MCO1 in optogenetic therapies, paving the way for future treatments aimed at restoring functional vision in patients with retinitis pigmentosa, Stargardt disease, and similar conditions.

### 2.2. Microbial Hyperpolarizing Opsins

#### 2.2.1. NpHR (Natronomonas Halorhodopsin)

The discovery of Natronomonas halorhodopsin (NpHR), a light-activated chloride pump, introduced a novel mechanism for optogenetic vision restoration through its ability to hyperpolarize neurons in response to yellow light, effectively silencing neural activity. Zhang et al. (2009) demonstrated NpHR’s utility for precisely controlled neuronal inhibition, setting the foundation for optogenetic applications where the hyperpolarization of retinal cells can restore the OFF pathway in degenerative retinal conditions [68]. This approach allowed for distinct ON and OFF signaling by coexpressing ChR2 and NpHR in retinal ganglion cells, thereby replicating naturalistic light responses within degenerated retinas [68]. To further enhance NpHR’s utility, an improved variant, eNpHR (enhanced NpHR), was engineered to prevent cellular aggregation and improve membrane localization by adding a signal peptide and an export sequence. These modifications increased the peak photocurrent and enabled robust, sustained hyperpolarization without adverse cellular effects. The optimized eNpHR allowed for stronger and more reliable inhibition, expanding its applicability in optogenetic interventions aimed at vision restoration [69]. Further studies advanced the application of eNpHR by demonstrating its potential in reactivating light-insensitive cone cells in RP models. eNpHR could substitute the native phototransduction pathway in degenerated cones, restoring light sensitivity and enabling the generation of OFF responses in photoreceptor-deficient retinas [70]. These findings underscore eNpHR’s potential to enable more naturalistic vision restoration by facilitating both ON and OFF pathways, offering an optogenetic strategy to restore contrast sensitivity and improve visual function in patients with advanced retinal degeneration.

#### 2.2.2. Jaws

Jaws, a red-shifted halorhodopsin developed by Chuong et al. (2014), offered substantial improvements in optogenetic inhibition through its ability to hyperpolarize neurons under red light, thus facilitating deeper tissue penetration with minimal phototoxicity [71]. Derived from Haloarcula salinarum, Jaws was engineered to produce photocurrents three times stronger than eNpHR. This enhanced efficacy was demonstrated in mouse models of retinitis pigmentosa, where Jaws successfully suppressed sensory-evoked activity in retinal ganglion cells, thereby mitigating the effects of photoreceptor degeneration and improving visual response reliability in treated subjects [71]. Subsequent studies explored the application of Jaws for optogenetic vision restoration in non-human primates. Jaws, when delivered intravitreally to foveal cones using a tailored AAV vector, restored light sensitivity in cone cells, producing robust, red-light-elicited hyperpolarization. The results demonstrated that Jaws could be successfully expressed in primate foveal cones, reinforcing its potential as a safe and effective tool for clinical translation in optogenetic vision restoration therapies [72].

## 3. Animal Opsins

### 3.1. OPN4 (Melanopsin)

The application of melanopsin (OPN4), a light-sensitive G-protein-coupled receptor naturally expressed in intrinsically photosensitive retinal ganglion cells, has introduced a pathway for restoring light sensitivity in degenerated retinas. In 2008, Lin et al. demonstrated that ectopic expression of melanopsin in retinal ganglion cells of rd1 mice led to a recovery of light-driven behavioral responses, such as the pupillary light reflex and light avoidance, under natural light conditions [73]. This study provided initial evidence that melanopsin could enable sustained, naturalistic responses in models of photoreceptor degeneration, highlighting its potential for clinical application [73]. Further studies on rd1 mice confirmed that human melanopsin expressed via AAV2/8 vectors could transiently restore light responses in photoreceptor-depleted retinas, indicating the feasibility of using melanopsin for short-term therapeutic interventions [74]. In end-stage retinal degeneration mice, De Silva et al. (2017) utilized subretinal delivery of human melanopsin, achieving stable visual function restoration up to 13 months post-treatment [75]. The study underscored the advantages of melanopsin’s intrinsic signal amplification, which enhances light sensitivity without requiring high-intensity light, a critical feature for patient safety and comfort [75].

### 3.2. Opto-mGluR6

To enhance signal specificity, Van Wyk et al. (2015) developed Opto-mGluR6, a chimeric protein combining the light-sensing domains of melanopsin with the ON bipolar cell-specific receptor mGluR6 [76]. This fusion enabled selective activation of the ON pathway under daylight conditions, providing a physiological compatibility that supports naturalistic visual signaling in blind retinas. By leveraging the signal amplification properties of the G-protein-coupled receptor pathway, Opto-mGluR6 achieved light sensitivity levels compatible with daylight, marking a substantial improvement over microbial opsins for clinical vision restoration [76]. A CRISPR-/Cas-mediated approach was introduced to enhance the specificity and efficiency of Opto-mGluR6 expression in retinal ON bipolar cells by targeting the endogenous GRM6 promoter. This advancement addressed previous limitations in cell-specific expression, which can lead to off-target effects and suboptimal therapeutic outcomes. Employing CRISPR/Cas systems for targeted knock-in demonstrated sustained functional restoration in mouse models of retinal degeneration, underscoring the potential of leveraging endogenous promoters to improve safety and precision in optogenetic therapies for vision restoration [77].

### 3.3. LiGluR (Light-Gated Ionotropic Glutamate Receptor)

The introduction of LiGluR (light-gated ionotropic glutamate receptor) provided a novel approach to vision restoration by utilizing an engineered mammalian ionotropic glutamate receptor that can be precisely activated and deactivated with light. LiGluR’s unique design incorporates a photoswitchable azobenzene molecule that allows the receptor to open and close upon exposure to specific wavelengths (380 nm for activation and 500 nm for deactivation). When delivered intravitreally via AAV2 vectors to retinal ganglion cells (RGCs) in retinal degeneration mice, LiGluR restored light sensitivity to RGCs, reinstating VEPs in the primary visual cortex and enabling behavioral responses such as the pupillary reflex and light avoidance [78].

### 3.4. Rho (Human Rod Opsin)

Human rhodopsin presents a promising approach to optogenetic vision restoration due to its high intrinsic light sensitivity, which enables activation under ambient light conditions. In murine models of advanced retinal degeneration, by using AAVs for gene delivery, the rod opsin can successfully induce expression in retinal ganglion and ON bipolar cells, enabling light responses including the detection of spatial and temporal visual cues, such as flicker and movement within naturalistic scenes. This suggests that rod opsin can achieve functional vision restoration under physiologically relevant conditions [79]. Human rhodopsin’s natural compatibility with human retinal structure minimizes immunogenicity risks. Additionally, the G-protein-coupled signaling of rod opsin offers amplified responses, allowing effective neural activation with minimal light exposure. The application of rod opsin as an optogenetic tool highlights a strategic shift toward leveraging endogenous proteins for vision restoration, offering a biologically harmonious and clinically adaptable solution for individuals with retinal degenerative diseases [79].

### 3.5. SNAG-mGluR2

The development of SNAG-mGluR2, a photoactivatable G-protein-coupled receptor engineered by chemically modifying mGluR2 (metabotropic glutamate receptor 2), added a significant new tool for optogenetic vision restoration. SNAG-mGluR2, which was equipped with a photoswitch to enable light-dependent hyperpolarization, selectively evoked OFF responses in RGCs in blind mice. When expressed in RGCs of the rd1 mouse model, SNAG-mGluR2 enabled these cells to produce robust, light-evoked OFF responses, allowing the mice to discriminate between patterns, such as parallel and perpendicular lines, as well as differences in line spacing. This capability signified a meaningful step toward restoring patterned vision in degenerated retinas [80]. A unique aspect of SNAG-mGluR2 lies in its reliance on G-protein signaling to modulate potassium channel activity, leading to hyperpolarization in response to light, which mimics natural OFF responses. This innovation permits the restoration of a critical aspect of visual processing, enabling differentiation between light patterns that mimic natural visual stimuli. Moreover, when combined with the ON-response-generating LiGluR, SNAG-mGluR2 coexpression led to a mix of ON, OFF, and ON–OFF responses across the RGC population, further enhancing visual acuity and enabling finer pattern recognition. This combinatorial strategy highlights SNAG-mGluR2’s therapeutic potential, as it not only restores light sensitivity but also supports more nuanced and naturalistic vision restoration [80].

### 3.6. MW-Opsin (Medium-Wavelength Cone Opsin)

The medium-wavelength cone opsin (MW-opsin) was developed as a promising optogenetic tool for vision restoration due to its high light sensitivity, adaptability to ambient light, and fast response kinetics. MW-opsin could overcome the limitations posed by slower, less adaptable opsins, such as rhodopsin. When expressed in retinal ganglion cells of the rd1 mouse model, MW-opsin enabled these cells to respond robustly to light under typical indoor conditions. Its faster kinetics and adaptive properties allowed MW-opsin to support both stationary and moving pattern discrimination, mirroring natural photoreceptor responses essential for visual processing [81]. This adaptive response, which permits effective functioning across a wide range of light intensities, represents a distinct advantage over previous opsins, making MW-opsin especially suitable for scenarios where light conditions fluctuate. MW-opsin restored natural behaviors in mice, such as object exploration in varied lighting, without requiring external amplification devices [81].

### 3.7. GHCR (Gleobacter–Human Chimeric Rhodopsin)

The development of GHCR (Gleobacter–human chimeric rhodopsin), a chimeric opsin that combines elements from Gloeobacter and human rhodopsins, represents a novel advancement in optogenetic strategies for vision restoration. GHCR enhanced light sensitivity by fusing segments of human rhodopsin with microbial rhodopsin, thereby leveraging the G-protein signaling pathways that are critical for physiological photoreception. This chimeric design enabled GHCR to achieve significant visual restoration in low-light environments, a substantial improvement over traditional microbial opsins, which typically require high-intensity light for activation. In mice, GHCR restores visual responses in retinal ganglion cells and transmits these signals to higher visual centers, as demonstrated by VEPs [82]. Behavioral tests in treated mice confirmed GHCR’s efficacy in naturalistic lighting, with the chimeric opsin supporting both day and night vision capabilities, mimicking the performance of native mammalian photoreceptors. Furthermore, GHCR exhibited stability over extended periods and conferred protective effects against retinal degeneration, offering a dual benefit of vision restoration and neuroprotection. This unique combination of properties positions GHCR as a promising candidate for clinical applications in patients with advanced retinal degenerations, providing functional vision restoration while mitigating disease progression [82].

## 4. Conclusions

**Figure 2:**
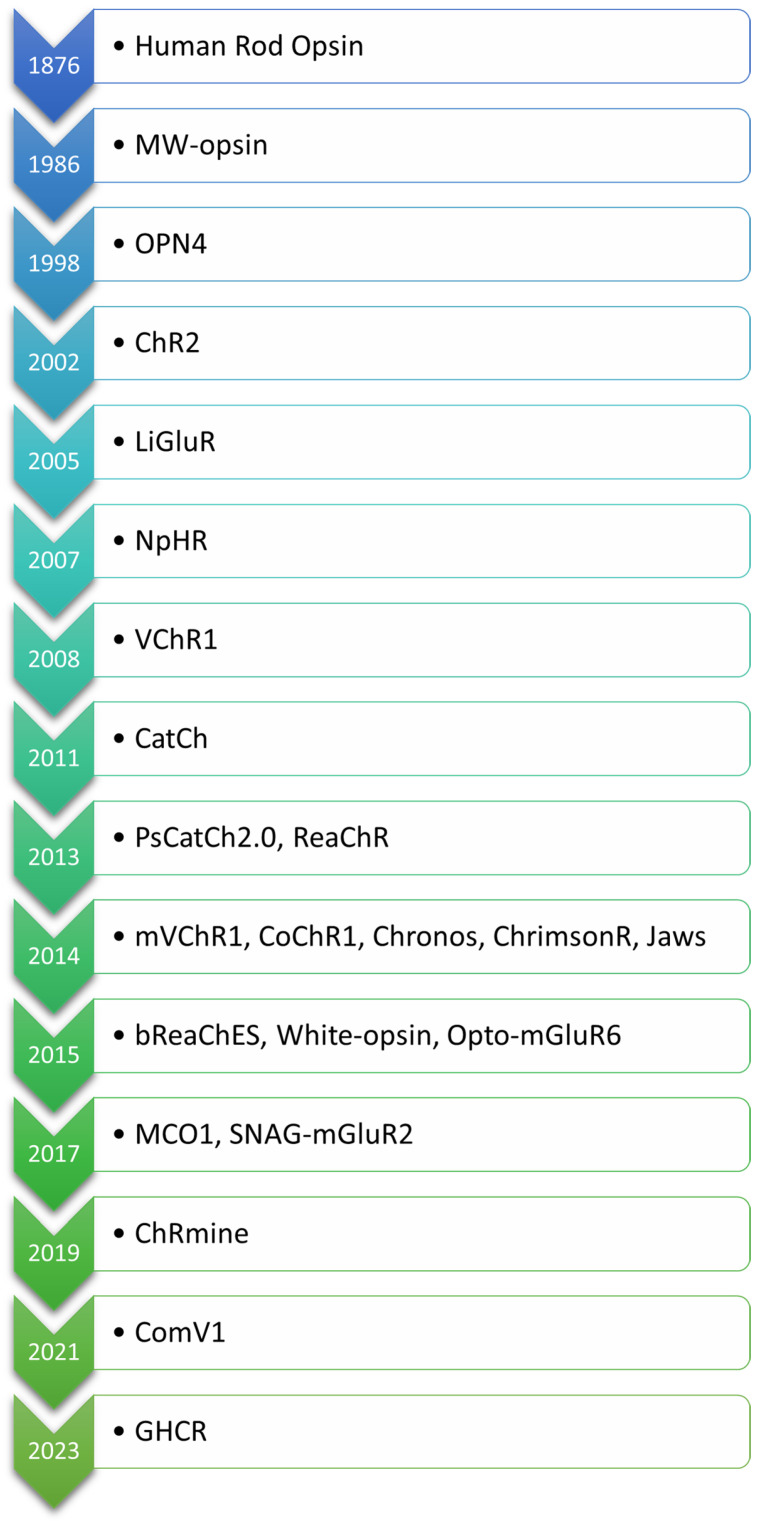
Chronological order of opsins used in optogenetic vision restoration, arranged according to the date of their first description.

The evolution of optogenetic approaches for vision restoration demonstrates remarkable advancements in engineering light-sensitive proteins to address the challenges posed by retinal degenerative diseases. The order in which the opsins are mentioned in the text is based on their first application in the context of optogenetic vision restoration, with related opsins listed together. For clarity, the opsins are arranged according to the date of their first description in Figure 2. An overview of key light-sensitive proteins used in optogenetic approaches for vision restoration, including their functions, activation mechanisms, and modifications aimed at enhancing light sensitivity and reducing phototoxicity, is presented in Table 1.

The field has progressed from pioneering microbial opsins like ChR2 to sophisticated variants such as ChrimsonR, ChRmine, and MCO1, which offer enhanced light sensitivity, red-shifted activation profiles, and reduced phototoxicity. These advancements have paved the way for translating optogenetic therapies from preclinical studies to clinical trials, with notable milestones like the first patient treated with ChrimsonR demonstrating real-world feasibility.

**Figure 3:**
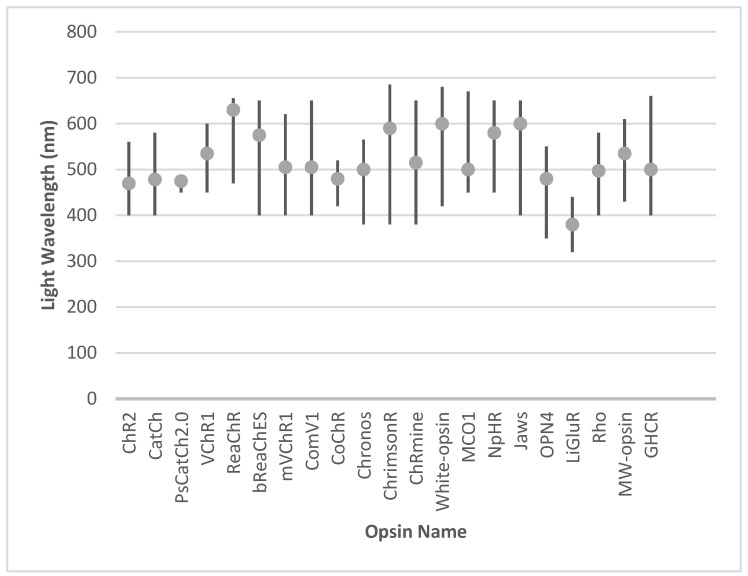
The activation range and optimal wavelength for the activation of individual opsins.

Innovations in red-shifted and broadband opsins, including ReaChR and white-opsin, have expanded the usability of optogenetics in ambient light conditions. Meanwhile, engineered opsins such as Chronos and CoChR deliver superior temporal resolution and precision, and hyperpolarizing tools like Jaws and NpHR complement these by enhancing contrast sensitivity and restoring naturalistic visual responses. The integration of animal-derived opsins, such as melanopsin and human rhodopsin, has provided biologically harmonious solutions, leveraging native signaling pathways. Additionally, chimeric designs like GHCR and signaling-specific innovations such as SNAG-mGluR2 further diversify the therapeutic toolkit, enabling both functional restoration and neuroprotection. The activation range and optimal wavelength for the activation of individual opsins are presented in Figure 3.

Despite the remarkable progress in optogenetic technologies, significant challenges remain that must be addressed to enable widespread clinical implementation. One of the foremost barriers lies in the safe and effective delivery of light to retinal tissues, particularly in advanced retinal degeneration where structural remodeling complicates light penetration. Structural remodeling, which includes the migration of retinal neurons, gliosis, and the disorganization of retinal layers, can scatter or absorb incoming light, reducing its intensity and precision before it reaches the target cells. This scattering effect diminishes the activation efficiency of opsins and can limit the restoration of vision. Current solutions, such as light-amplifying goggles and optical implants, are promising but face practical limitations, including bulkiness, cost, and adaptability to real-world environments. Furthermore, the long-term safety of sustained opsin expression raises concerns about immunogenicity, potential off-target effects, and cumulative phototoxicity, particularly in patients requiring prolonged treatment. Clinical variability, influenced by factors such as residual retinal cell viability and the extent of disease progression, also poses a challenge to achieving consistent therapeutic outcomes across diverse patient populations.

The future of optogenetics lies in refining the technologies for widespread clinical application. Ongoing efforts to enhance light sensitivity, minimize phototoxicity, and improve cell specificity will be crucial. Additionally, the convergence of optogenetics with adjunctive therapies, such as retinal prosthetics, stem cell treatments, and gene therapies, promises to optimize outcomes for patients with retinal degenerations. The interplay between these modalities requires further investigation to optimize efficacy and avoid potential conflicts.

Collectively, the developments highlight the transformative potential of optogenetics in vision restoration. As research continues to address existing limitations and enhance efficacy, optogenetic therapies are poised to revolutionize the management of retinal degenerative diseases, offering new hope for millions of individuals affected by vision loss worldwide.

## Figures and Tables

**Table 1:** Overview of light-sensitive proteins used in optogenetic approaches for vision restoration. This table presents key light-sensitive proteins utilized in optogenetics for vision restoration in patients with retinal degenerations. These light-sensitive proteins are categorized by their function, including depolarization and hyperpolarization, as well as activation mechanisms, such as ion channels and G-protein-dependent pathways. Additionally, modified proteins with enhanced light sensitivity or efficiency compared to their natural counterparts are highlighted. Red-shifted opsins are also noted for their lower phototoxicity risk, which is particularly advantageous for vision therapy. All listed opsins hold significant importance in the advancement of optogenetics, offering potential tools for restoring visual function by enabling selective activation of various retinal cell populations with specific light wavelengths.

Light-Sensitive Protein	Source	Function	Optimal Light Wavelength (nm)	Key Properties	Citations
ChR2 (Channelrhodopsin-2)	Chlamydomonas reinhardtii (algae)	Depolarization (light-gated non-selective cation channel)	~460	First opsin utilized in optogenetic vision restoration	Bi et al. (2006) [25], Tomita et al. (2007) [26], Lagali et al. (2008) [27], Tomita et al. (2010) [28], Doroudchi et al. (2011) [29], Macé et al. (2015) [30]
CatCh (Calcium-Translocating Channelrhodopsin)	Modification of ChR2	Depolarization (light-gated non-selective cation channel)	~478	Improved Ca^2+^ permeability, leading to increased light sensitivity compared to ChR2	Kleinlogel et al. (2011) [31], Chaffiol et al. (2017) [32]
PsCatCh2.0 (Platymonas subcordiformis Channelrhodopsin 2.0)	Modification of PsChR from Platymonas (Tetraselmis) subcordiformis	Depolarization (light-gated non-selective cation channel)	~475	Improved Ca^2+^ and Na^+^ conductance and kinetics compared to CatCh	Govorunova et al. (2013) [33], Chen et al. (2022) [34]
NpHR (Halorhodopsin)	Natronomonas pharaonis (archaea)	Hyperpolarization (light-gated Cl^−^- pump)	~580	First hyperpolarizing opsin utilized in optogenetic vision restoration	Zhang et al. (2009) [35]
eNpHR (Enhanced Halorhodopsin)	Modification of NpHR	Hyperpolarization (light-gated Cl^−^- pump)	~580	Improved distribution within the cell, with stronger localization in the cell membrane compared to NpHR	Gradinaru et al. (2008) [69], Busskamp et al. (2010) [70]
VChR1 (Volvox Channelrhodopsin-1)	Volvox carteri (algae)	Depolarization (light-gated non-selective cation channel)	~535	Red-shifted microbial opsin	Zhang et al. (2008) [35], Kianianmomeni et al. (2009) [36]
ReaChR (Red-Shifted Channelrhodopsin)	Modification of VChR1	Depolarization (light-gated non-selective cation channel)	~630	Modified red-shifted microbial opsin; functions effectively over a broad spectrum of light wavelengths	Lin et al. (2013) [37], Sengupta et al. (2016) [38], Ferrari et al. (2020) [39]
bReaChES	Modification of ReaChR	Depolarization (light-gated non-selective cation channel)	~575	Modified red-shifted microbial opsin; functions effectively over a broad spectrum of light wavelengths; exhibits high light sensitivity and temporal resolution	Rajasethupathy et al. (2015) [40], Too et al. (2022) [41]
mVChR1 (Modified VChR1)	Chimeric protein combining Volvox channelrhodopsin-1 and Chlamydomonas channelrhodopsin-1	Depolarization (light-gated non-selective cation channel)	~505	Functions effectively over a broad spectrum of light wavelengths	Tomita et al. (2014) [42]
ComV1 (ex3mV1)	Modification of mVChR1	Depolarization (light-gated non-selective cation channel)	~ 505	Improved light sensitivity compared to mVChR1	Watanabe et al. (2021) [43]
H172A Mutant of ComV1	Modification of ComV1	Depolarization (light-gated non-selective cation channel)	~500	Improved kinetics compared to ComV1	Hatakeyama et al. (2023) [44]
Jaws	Modified opsin from Haloarcula salinarum (Halobacteriaceae)	Hyperpolarization (light-gated Cl^−^- pump)	~600	Red-shifted microbial hyperpolarizing opsin	Chuong et al. (2014) [71], Khabou et al. (2018) [72]
CoChR (Chloromonas oogama Channelrhodopsin)	Chloromonas oogama	Depolarization (light-gated non-selective cation channel)	~480	Microbial opsin with improved light sensitivity	Klapoetke et al. (2014) [45], Schild et al. (2015) [46], Shemesh et al. (2017) [47]
CoChR-LC	Modification of CoChR	Depolarization (light-gated non-selective cation channel)	~480	Improved light sensitivity compared to CoChR	Ganjawala et al. (2019) [48]
CoChR-3M	Modification of CoChR	Depolarization (light-gated non-selective cation channel)	~480	Improved light sensitivity compared to CoChR	Ganjawala et al. (2019) [48]
Chronos (ShChR, Stigeoclonium Helveticum Channelrhodopsin)	Stigeoclonium helveticum (algae)	Depolarization (light-gated non-selective cation channel)	~500	Microbial opsin with improved kinetics	Klapoetke et al. (2014) [45], Yan et al. (2023) [49]
ChrimsonR (CnChR1, Chlamydomonas Noctigama Channelrhodopsin 1)	Chlamydomonas noctigama (algae)	Depolarization (light-gated non-selective cation channel)	~590	Red-shifted microbial opsin	Klapoetke et al. (2014) [45], Cheong et al. (2018) [50], Chaffiol et al. (2021) [51], Gauvain et al. (2021) [52], McGregor et al. (2022) [54]
ChRmine	Rhodomonas lens (algae)	Depolarization (light-gated non-selective cation channel, with the structure partially resembling a cation pump)	~515	Red-shifted microbial opsin; high light sensitivity; broad activation spectrum	Marshel et al. (2019) [55], Kishi et al. (2022) [56]
hsChRmine	Modification of ChRmine	Depolarization (light-gated non-selective cation channel, with the structure partially resembling a cation pump)	~515	Improved temporal resolution compared to ChRmine	Kishi et al. (2022) [56], Bansal et al. (2024) [57]
frChRmine	Modification of ChRmine	Depolarization (light-gated non-selective cation channel, with the structure partially resembling a cation pump)	~585	Enhanced red-light sensitivity compared to ChRmine	Kishi et al. (2022) [56], Bansal et al. (2024) [57]
rsChRmine	Modification of ChRmine	Depolarization (light-gated non-selective cation channel, with the structure partially resembling a cation pump)	~510	Slightly red-shifted activation range compared to ChRmine	Kishi et al. (2022) [56], Bansal et al. (2024) [57]
White-opsin	Fusion of ChR2, C1V1, and ReaChR opsins	Depolarization (complex mechanism based on channel properties of the fused proteins)	~600	Broad spectral excitability across the visible spectrum	Batabyal et al. (2015) [58]
MCO1 (Multi-Characteristic Opsin 1)	Designed in silico and laboratory-produced	Depolarization (complex mechanism)	~500	Exhibits high light sensitivity and functions effectively over a broad spectrum of light wavelengths	Wright et al. (2017) [59], Wright et al. (2017) [60], Batabyal et al. (2021) [61], Boyer et al. (2023) [64], Gonzalez et al. (2023) [66], Mahajan (2024) [67], Batabyal et al. (2024) [62], Batabyal et al. (2024) [63], Ho (2024) [65]
OPN4 (Melanopsin)	Vertebrates (including Homo sapiens)	Depolarization (G-protein-dependent pathway)	~480	First vertebrate opsin utilized in optogenetic vision restoration	Lin et al. (2008) [73], Liu et al. (2016) [74], de Silva et al. (2017) [75]
Opto-mGluR6	Chimeric protein combining the light-sensing domains of OPN4 with the ON bipolar cell-specific metabotropic glutamate receptor mGluR6	Depolarization (G-protein-dependent pathway)	~479	Chimeric protein reaching increased light sensitivity	Van Wyk et al. (2015) [76]
LiGluR (Light-Gated Ionotropic Glutamate Receptor)	Engineered mammalian ionotropic glutamate receptor	Depolarization (light-gated non-selective cation channel)	Bistable photoswitch (380 nm opens the channel; 500 nm closes it)	Utilizes a reversible photoswitch for activation and deactivation	Caporale et al. (2011) [78]
Rho (Human Rhodopsin)	Homo sapiens	Depolarization (G-protein-dependent pathway)	~497	Human opsin expressing high light sensitivity	Cehajic-Kapetanovic et al. (2015) [79]
SNAG-mGluR2 (Photoswitch-Charged Metabotropic Glutamate Receptor 2)	Fusion of mammalian metabotropic glutamate receptor 2 with a SNAP photoswitch	Hyperpolarization (G-protein-dependent pathway)	~445	Evokes OFF responses to light	Berry et al. (2017) [80]
MW-opsin (Medium-Wavelength Cone Opsin)	Vertebrates (including Homo sapiens)	Depolarization (G-protein-dependent pathway)	~535	Exhibits light sensitivity comparable to rhodopsin, while demonstrating ten-times-faster kinetics	Berry et al. (2019) [81]
GHCR (Gleobacter–Human Chimeric Rhodopsin)	Chimeric protein replacing parts of Gloeobacter (cyanobacteria) rhodopsin with parts of human rhodopsin	Depolarization (light-gated non-selective cation channel activating G-protein-dependent pathway)	~500	Microbial–human opsin chimera; high light sensitivity and increased adaptation to light intensity changes compared to microbial and vertebrate opsins	Katada et al. (2023) [82]

## Data Availability

All relevant data are presented within this paper.
